# Optimization of the imaging protocol of an X-ray CT scanner for evaluation of normoxic polymer gel dosimeters

**DOI:** 10.4103/0971-6203.26692

**Published:** 2006

**Authors:** Brindha Subramanian, Paul B. Ravindran, Clive Baldock

**Affiliations:** Department of Radiotherapy, Christian Medical College, Vellore, Tamil Nadu, India; * Department of Institute of Medical Physics, School of Physics, University of Sydney, Sydney, Australia

**Keywords:** Normoxic polymer gels, X-ray CT, imaging protocol

## Abstract

X-ray computer tomography (CT) has previously been reported as an evaluation tool for polymer gel (PAG) dosimeters. In this study, the imaging protocol of a Siemens Emotion X-ray CT scanner was optimized to evaluate PAGAT normoxic gel dosimeters. The scan parameters were optimized as 130 kV and 150 mA with a slice thickness of 3 mm for smaller fields and 5 mm for larger fields of irradiation. The number of images to be averaged to reduce noise to an acceptable level was concluded to be 25. It was also concluded that the total monomer concentration required is 7% with 10 mM THP to obtain a maximum change in CT number at dose levels up to 15 Gy for evaluation with X-ray CT. Optimal scan parameters may vary with X-ray CT scanner. Hence the imaging protocol of each scanner to be used for evaluating polymer gels requires individual optimization for the purposes of gel dosimetry evaluation.

In 1984 Gore *et al*. proposed a method for determining the spatial distribution of radiation dose in tissue equivalent phantom using nuclear magnetic resonance imaging.[1] Since then studies have been undertaken to investigate the feasibility of using gel dosimetry as a 3-D dosimetry system in radiation oncology.[2–4]

In Fricke gel dosimeters, ferrous ions (Fe^2+^) are converted to ferric ions (Fe^3+^) due to ionizing radiation. Fricke gels however have a major limitation due to loss of spatial integrity due to the diffusion of ferric ions.[5]

In 1993, a polymer gel dosimeter based upon polymerization of acrylamide (AA) and N'N'- methylene-bis-acrylamide (BIS) monomers infused in an aqueous gel matrix was developed.[6] This polymer gel dosimeter formulation, often described as a polyacrylamide gel or PAG[7] is normally manufactured in an oxygen free or hypoxic atmosphere due to the inhibitory effect of oxygen on the polymerization process.[8–12]

Normoxic polymer gel dosimeters[13] are produced, stored and irradiated in a normal or normoxic atmosphere. The first suggested normoxic polymer gel dosimeter, known as MAGIC, an acronym for Methacrylic acid, Ascorbic acid, Gelatin Initiated by Copper was based upon the polymerization of methacrylic acid infused with copper (II) sulphate and the antioxidant ascorbic acid in a gel matrix. Subsequently, a number of other normoxic polymer gel dosimeter formulations were investigated including MAGAS.[14] Tetrakis (hydroxy methyl) phosphonium chloride (THP) was found to be a very effective antioxidant.[11] Polymer gel dosimeter compositions containing the THP antioxidant include MAGAT (methacrylic acid, gelatin and tetrakis)[15] and PAGAT (polyacrylamide, hydroquinone, gelatin and tetrakis).[16]

To date magnetic resonance imaging (MRI) has been most extensively used for the evaluation of absorbed dose distributions in polymer gel dosimeters (2-4 and references therein). Other evaluation techniques such as optical tomography,[17] vibrational spectroscopy,[18] X-ray computerized tomography (CT)[19] and ultrasound[20] have also been investigated.

X-ray CT has been used as an evaluation tool to measure dose distributions of irradiated polymer gel dosimeters.[21] The resulting images have CT numbers or Hounsfield units (H) related to linear attenuation data, which is directly linked to physical density changes of irradiated polymer gel dosimeters. The post-irradiation changes in the linear attenuation coefficient, μ have been evaluated.[22,23] Technical considerations for evaluating PAG gels with X-ray CT has already been reported.[24] The feasibility of using X-ray CT has further been evaluated for normoxic polymer gel dosimeters by studying radiological attenuation properties as well as the dose response characteristics of normoxic gel dosimeters.[25–27] In the current work, the imaging protocol of a Siemens Emotion single slice spiral CT scanner was optimized for evaluating PAGAT polymer gels in addition to the optimization of monomer concentration for obtaining an optimum change in CT number with radiation dose.

## Materials and Methods

### Preparation of PAGAT gel dosimeter

Normoxic PAGAT gel dosimeters were prepared under normal atmospheric conditions as per the recipe previously published.[16,28] For preparing the PAGAT gel dosimeter for optimization the imaging protocol of the CT scanner, 5% by total weight of gelatin was mixed with 89% distilled water and allowed to soak for 10 minutes before heating to 50°C in a conical flask. Once the temperature reached 50°C, the gel mixture was removed from the heater and 3% BIS was added, stirred thoroughly and allowed to dissolve. After dissolving the BIS, 3% acrylamide was added and allowed to dissolve. When the dosimeter was ready to be poured into the vials, 10 mM of THP was added and mixed thoroughly. The gel was poured into two 200 ml plastic bottles.

In order to optimize the concentration of monomers for evaluation with X-ray CT, the total concentration of monomer was varied. The concentration of BIS and AA were varied from 3 to 4.5% by total weight of gel dosimeter required. Four sets of gels with concentration of BIS as 3, 3.5, 4 and 4.5% by total weight of required gel dosimeter and concentration of AA as 3, 3.5, 4 and 4.5% by total weight respectively of the gel required were prepared. The PAGAT gel dosimeter was then poured into cylindrical plastic vials of height 5.6 cm and diameter 2.4 cm for optimization of monomer concentration and refrigerated.

### Irradiation of the PAGAT gel dosimeters

The gels that were in the plastic bottles were left un-irradiated to study the variation of standard deviation of averaged CT images. The PAGAT gel dosimeters in the vials were irradiated to 5, 10 and 15 Gy at d_max_ with 6 MV photons using a field size of 30 × 30 cm^2^ from a Siemens Mevatron MD linear accelerator at 100 cm SSD using parallel opposed fields to obtain a uniform dose in the vials. The irradiated gels were left in the refrigerator for 24 hours before imaging assuming the polymerization due to irradiation is completed since it has been considered that the post-irradiation polymerization was essentially completed after approximately 12 hours post-irradiation.[12]

### Imaging

The irradiated gels were placed horizontally on the flat couch top of the Siemens Somatom CT scanner for imaging. To determine the number of images to be averaged, the imaging parameters of 80 kV and 130 kV were used with maximum mA and the un-irradiated sample of the PAGAT gel dosimeter in the plastic bottle was imaged 50 times at the same scan position. For the optimization of concentration of monomers, the highest available tube voltage of 130 kV and tube current of 110 mA were used with an imaging time of 0.8 s. For optimizing the imaging protocol of the CT scanner all the scan parameters such as the tube voltage, tube current and slice thickness were varied.

### Evaluation

The CT images were transferred to a PC and evaluated with modified software coded in MATLAB™.[29] The software enabled averaging of images obtained at the same scan position, marking circular regions of interest (ROI) of varying diameter within the circumference of the bottles or vials, conversion of data to standard Hounsfield units (H) or CT number in the selected ROI and calculation of standard deviation of H within the ROI. The variation in two factors namely the standard deviation in CT number (σN_CT_) and changes in CT number (ΔN_CT_) were evaluated.

### Optimization of number of images to be averaged

The CT images of the PAGAT gel had a low signal to noise ratio (SNR) and this has been found to improve by image averaging (Hilts *et al*., 2000). It is time consuming to obtain 50 scans in each slice while validating radiotherapy treatment plans in a clinical set up. Hence a study was carried out to obtain an optimal number of scans at the same scan position to reduce the noise as well as to be less time consuming.

The un-irradiated sample of the PAGAT gel dosimeter in the plastic bottle was imaged 50 times at the same scan position with the following settings: tube voltage - 80kV and 130 kV, tube current 150 mA, exposure time 0.8 s and slice thickness 5 mm. The data was transferred to a PC and evaluated. Different number of images up to 50 images was averaged and upon which a circular ROI was drawn. The ROI was 292.58 mm^2^ in 749 pixels with each pixel size of 0.3906 mm^2^. The CT numbers were extracted by taking the mean ROI value on each of the averaged image and the standard deviation in CT number (σN_CT_) for all the averaged images over the selected ROI was calculated.

An investigation on the effect of image averaging on the CT number of the irradiated gel was also carried out. One set of plastic vials irradiated to doses of 5, 10 and 15 Gy was imaged 50 times at the same scan position. The images were transferred to a PC and evaluated.

The SNR for different number of images averaged was calculated for different number of images averaged from $SNR=\frac{N_{CT}\left(irradiatedgel\right)\text{- }N_{CT}\left(un\text{-}irradiatedgel\right)}{\sigma }$

where s is the standard deviation in the averaged CT number obtained from the selected ROI within the vial of the un-irradiated PAGAT gel dosimeter.

### Optimization of scanner parameters

The first step in optimizing the scan parameters was to optimize the tube voltage and tube current at which the dosimeter was to be imaged. For this, the un-irradiated PAGAT gel dosimeter was imaged with the available tube voltages of 80 kV, 110 kV and 130 kV with minimum and maximum available tube current for each tube voltage. The imaging was carried out with slice thickness of 1, 2, 3, 5 and 10 mm respectively. Circular regions of interest of area 163 mm^2^ over 221 pixels were drawn at the central region of the vials in the averaged image. The software calculates the CT numbers by taking the mean of the CT numbers in each pixel in the ROI selected. The average CT number and the standard deviation in CT number (σN_CT_) for all the averaged images over the selected ROI were obtained.

### Optimization of monomer concentration

For optimization of monomer concentration, the maximum change in CT number (ΔN_CT_) with absorbed dose was studied. The PAGAT gel for this study was prepared, irradiated and imaged as discussed in sections 2.1, 2.2 and 2.3. The images were transferred to a PC and analyzed. The average of 50 images for each dose was calculated using the software. Circular regions of interest of area 163 mm^2^ over 221 pixels were drawn at the central region of the vials in the averaged image and the CT numbers were obtained. The change in CT number with radiation dose was calculated by subtracting the CT number of the irradiated gel from that of the un-irradiated gel.

## Results and Discussion

### Optimization of the imaging protocol of the X-ray CT scanner

**Figure 1:**
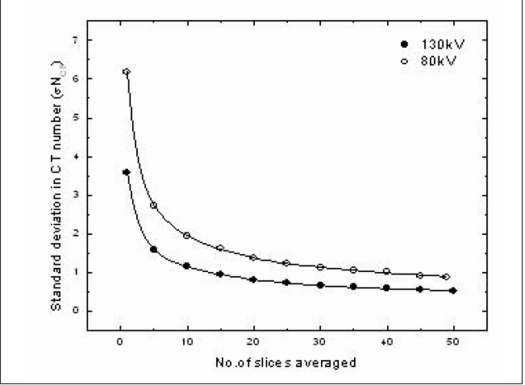
Variation in standard deviation of CT number with change in the number of images averaged

Figure 1 shows the variation in standard deviation (σN_CT_) obtained over a region of interest of the averaged images for both the lowest and highest tube voltage available in the X-ray CT scanner for which the imaging protocol was optimized. The σN_CT_ decreased from 6.19 H to 1.22 H for a tube voltage of 80 kV and from 3.6 H to 0.7 H for 130 kV when the number of images averaged was increased from 1 to 25. This shows that the noise due to photon counting statistics can be reduced by image averaging. After this point, the decrease in σN_CT_ was found to be 0.3 H when the number of images averaged increased from 25 to 50. The resulting total noise of 0.5 H when 50 images were averaged compares with the value of 0.6 H obtained by Trapp *et al*., 2001. This is a known effect in CT imaging and is due to factors such as reconstruction noise, electronic noise and CT number quantization. At 80 kV the HU for the normoxic polymer gel dosimeters was lower than that for 130 kV indicating that the HU changes with tube potential. This was to be expected, as linear attenuation of the normoxic polymer gel dosimeter is energy dependent. Moreover, the load on the X-ray tube is directly proportional to the number of images averaged. Since the variation in σN_CT_ was minimal when the number of images averaged increased from 25 to 50, it was decided to average 25 images to reduce the noise in the image and also keep the load on the X-ray tube at a lesser level when compared to averaging a large number of images.

**Figure 2:**
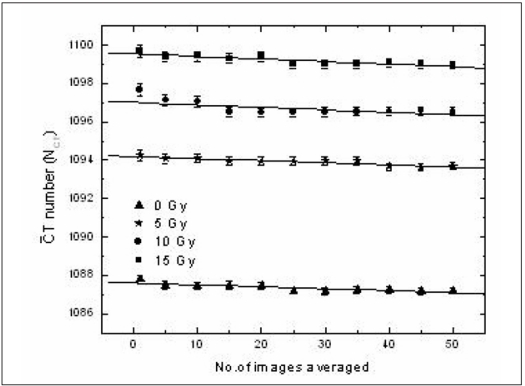
Variation in CT number (NCT) with the number of images averaged

**Figure 3:**
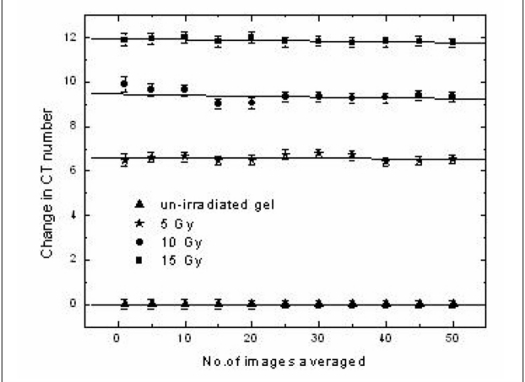
Variation in change in CT number (ΔN_CT_) with the number of images averaged

Figure 2 represents the variation in CT number with number of images averaged. The CT number was found to decrease as the number of images averaged increased. The maximum variation observed in CT numbers due to image averaging was 1 H for a dose of 15 Gy while the decrease was 0.5 H for lesser doses. This decrease is due to the variation in CT numbers in the pixels within the ROI. But when the variation in change in CT number (ΔN_CT_) with number of images averaged was calculated as shown in Figure 3, it was found that the ΔN_CT_ remained unchanged over the number of images averaged for all doses. The decrease in CT number observed in Figure 2 was removed when the difference between the irradiated and un-irradiated samples were calculated. From this it was concluded that the image averaging reduces the image noise alone without causing changes in the CT number.

**Figure 4:**
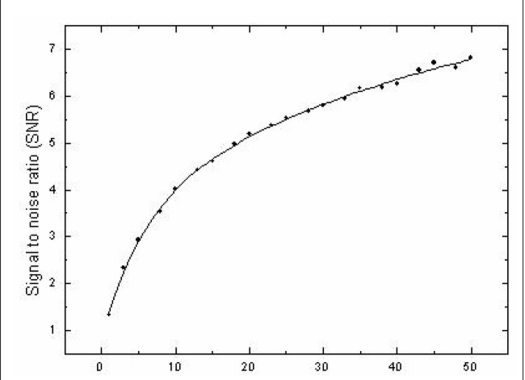
Variation in signal to noise ratio (SNR) with the number of images averaged

The variation in signal to noise ratio with increase in number of images averaged is shown in Figure 4. The SNR was found to increase asymptotically with averaged images as expected since theoretically the SNR increases by √*N* where N is the number of images averaged. The limitations in increasing the number of images in a single scan position are the load to the X-ray tube with increase in number of scans, polymerization in the gel phantom due to the radiation dose contribution during the scan as the number of images are increased and the time limitation in the availability of the an X-ray CT scanner in a clinical radiotherapy department. Obtaining a pre-irradiation scan and subtracting it from the post-irradiation scan could be used to correct the radiation dose contribution to the gel phantom during evaluation by X-ray CT. However, only decreasing the number of images averaged could decrease the load on the scanner as well as the time consumed on the scanner for imaging. From Figure 1 it was observed that the σN_CT_ decreased substantially when the number of images averaged increased from 1 to 25. The decrease was found to be less than 1 H for further increase in the number of images averaged. Hence the optimal number of images to be averaged was concluded as 25 to result in a desired SNR.

**Figure 5:**
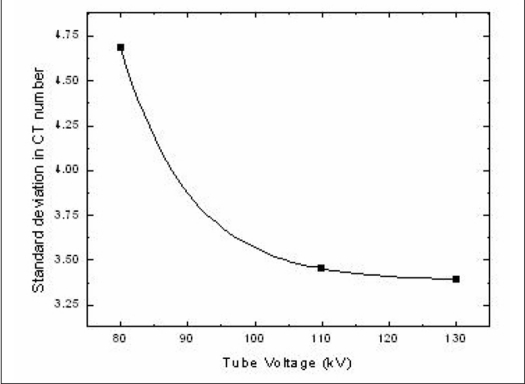
Effect of variation in tube voltage on the standard deviation of CT number in a circular region of interest selected

It has been shown that CT imaging technique affects the noise levels and these could be decreased by increasing tube voltage (kV), tube current (mA) and slice scan time (s).[24] Figure 5 showing the variation of σN_CT_ with tube voltage followed exponential decay pattern with decrease in image noise level with increase in tube voltage. The σN_CT_ decreased by 72% when the tube voltage increased from 80 kV to 130 kV. This reduction is found to be higher than the 60% reduction in doubling the tube voltage reported by Hilts *et al*., 2005. It was observed that the σN_CT_ decreased steeply by 1.23 H when the tube voltage increased from 80 kV to 110 kV. But beyond 110 kV, when the tube voltage was further increased to 130 kV, the change in standard deviation was only 0.6 H. σN_CT_ was found to be greater at lower tube voltages when compared to higher tube voltages as the maximum number of photons reaches the detector at the highest tube voltage providing a better SNR than the lower voltages characterized by lower photon emission.[30,^31^] Hence the optimum tube voltage at which the PAGAT polymer gel dosimeter is to be imaged was selected at the highest tube voltage of 130 kV available on the scanner being studied in spite of the increase in load to the tube taking into consideration the amount of noise in the images at a lower tube voltage of 80 kV.

**Figure 6:**
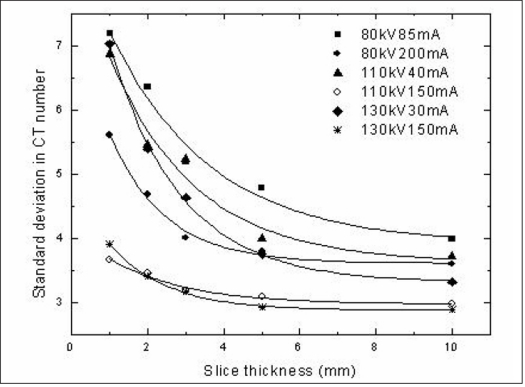
Variation in standard deviation in CT number with slice thickness for different combinations of tube voltage and tube current

Figure 6 shows σN_CT_ for different tube voltage and tube current combination with different slice thickness. All the curves followed the exponential decay pattern following the equation *Y = Y_0_ +Ae^x/t^* where x is the slice thickness in mm and Y is σN_CT_. However the standard deviation σN_CT_ was found to be minimal for two curves, one with a tube voltage of 110 kV and tube current of 150 mA and the other with a tube voltage of 130 kV and tube current of 150 mA. The σN_CT_ for the lower tube voltage of 110 kV was found to be lesser than that of the higher tube voltage of 130 kV for the lowest slice thickness of 1 mm. But beyond 1 mm, the σN_CT_ was more than that of 130 kV. Similarly, the standard deviation in CT number (σN_CT_) was found to be the maximum for a slice thickness of 1 mm and decreased with increase in slice thickness. While the variation in σN_CT_ was 0.6 H between 1 mm and 2 mm slice thickness, the change was found to be 0.2 H for further increase in slice thickness. There was a minimal change σN_CT_ as slice thickness increased from 5 mm to 10 mm. However while verifying dose distributions with dose gradients a lesser slice thickness is preferable. Therefore it was decided to use a 3 mm slice thickness for verification of small fields and 5 mm slice thickness for verification of larger fields.

### Optimization of monomer concentration

**Figure 7:**
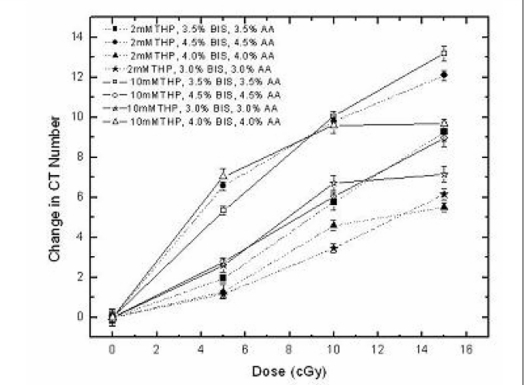
Dose response of the PAGAT gel dosimeter for different monomer concentrations with 2 mM THP and 10 mM THP

The dose response of the PAGAT polymer gel with 2 mM THP and 10 mM THP with various concentrations of BIS and acrylamide is shown in Figure 7. For 2 mM THP, the change in CT number was found to be 12 H for a dose of 15 Gy for the total monomer concentration of 9% (4.5% BIS and 4.5% Acrylamide) when compared to other concentrations. The 7% total concentration of monomers was 9 H for a dose of 15 Gy while the others had a lower change in CT numbers when compared to 7 and 9% total monomer concentration.

With 10 mM concentration of THP, the change in CT number was 13 H for a dose of 15 Gy for a total monomer concentration of 7% (3.5% BIS, 3.5% Acrylamide). The 9% total monomer concentration had the lowest change in CT number of 9 H for the dose of 15 Gy while the other concentrations had a lesser change in CT number when compared to 7% total monomer concentration.

From these observations a comparison of dose response between the total concentrations of 7 and 9% was investigated. It was found that with 7% total monomer concentration and 10 mM THP, the dose response curve was linear up to 10 Gy, with a maximum difference of 13 H for the dose of 15 Gy. For 9% total monomer and 2 mM THP, the linearity of dose response was observed till 5 Gy with a maximum difference of 12 H for 15 Gy. Hence, it was decided to optimize the total monomer concentration as 7% with 10 mM THP with a higher linear dose range for evaluating the PAGAT with X-ray CT scanner.

## Conclusion

The imaging protocol of the Siemens Emotion X-ray CT scanner was optimized to evaluate PAGAT normoxic gel dosimeters. Since the change in sN_CT_ was minimal beyond 25 images at the same scan position, the number of images to be averaged to reduce sN_CT_ in a region of interest thus reducing the noise was concluded to be 25. The scan parameters were optimized at the highest available tube voltage of 130kV and highest available tube current of 150mA to enable the maximum number of photons to reach the detector thereby reducing the noise in the image. The optimal slice thickness was determined to be 3 mm while evaluating small fields of irradiation and 5 mm for larger fields. The total concentration of monomers was optimized at 7 % with 10 mM THP to obtain a maximum change in CT number (DN_CT_) with absorbed dose for evaluating with X-ray CT. It has been shown that X-ray CT can be used as an evaluation tool for the PAGAT polymer gel dosimeter. Optimal scan parameters may vary with X-ray CT scanner. Hence each scanner to be used for evaluating polymer gels requires individual optimization for the purposes of gel dosimetry evaluation.
